# A retrospective review of the management of endoscopic sinus surgery (ESS) and frequency of concurrent adjunctive septoplasty

**DOI:** 10.1007/s11845-025-03997-2

**Published:** 2025-07-24

**Authors:** Karl Finucane, Séamus Boyle, Michael Fitzsimons, Michael Colreavy

**Affiliations:** Otolaryngology/Head and Neck Department, Mater Misericordiae University Hospital, Dublin 7, Ireland

**Keywords:** Concurrent septoplasty, Endoscopic sinus surgery, Septoplasty, Sinusitis

## Abstract

**Background:**

Septoplasty is indicated for nasal obstruction secondary to septal deviation. It is concurrently performed in up to 27% of endoscopic sinus surgery (ESS) to improve access.

**Aims:**

We aimed to determine the frequency and indications of adjuvant concurrent septoplasty in ESS.

**Methods:**

A retrospective chart review of patients who underwent ESS was performed. Patients included in the chart review were those who had ESS for any indication performed between 01/01/2022 to 31/12/2023 by two consultants ENT surgeons operating in The Mater Misericordiae University Hospital. Charts were examined to appreciate if septoplasty was performed, and consent was obtained prior to theatre. If the case was a revision surgery or if revision surgery was carried out after the initial procedure this was also noted.

**Results:**

In total, 84 patients underwent 90 cases of endoscopic sinus surgery were examined of which 6 cases were revision surgeries. Septoplasty was performed in 6 (6.6%) of these cases. Those who underwent adjuvant septoplasty were identified in the OPD. Factors for concomitant adjuvant septoplasty included a septal spur and caudal septal deviation.

**Conclusion:**

In our series we found that less than 10% of ESS required septoplasty and this should be considered during the consent process.

## Introduction

The concept of endoscopic sinus surgery (ESS) evolved primarily out of the detailed work of Messerklinger in evaluating mucociliary clearance patterns and endoscopic changes within the osteomeatal complex (OMC) and in incorporating computed tomography (CT) to image the ethmoid sinuses [1]. The principles of ESS were first published in 1985, and the first two courses on endoscopic intranasal surgery were held at the Johns Hopkins Hospital in the same year [2, 3]. Following this, there was rapid growth of both interest in and utilisation of endoscopic intranasal surgery.

ESS targets sinus pathology and is the gold standard for the surgical management of disease such as chronic rhinosinusitis (CRS) where success rates range from 76 to 98% [4, 5]. The boundaries of ESS are continually expanding with technological advances. At this point, the indications of ESS have surpassed the field of CRS. The application of this procedure marked its place in the management of sinus tumours and pathologies beyond the sinuses [6].

The notion behind ESS may seem straightforward, but due to anatomical variability and the broad range and severity of diseases addressed by ESS, every case remains a challenge. Common failure factors associated with ESS include poor surgical technique and a poor surgical field and visualisation [7]. Pre-operative planning for sinus surgery is the crucial step to obtain optimal results and to avoid all possible complications [6]. This is of particular importance in revision ESS [8].

Although modern endoscopes have reduced in size and surgical techniques improved since the days of Messerklinger, there can still be difficulties accessing the nasal sinuses due to obstruction, primarily from pathology of the nasal septum [9].

The nasal septum though primarily a support structure of the nose is also important in nasal physiology. Where there is anatomic anomalies of the nasal septum, this can lead to airway obstruction which can cause secondary reactive mucosal engorgement leading to sinus disease [10].

Septoplasty is one of the most common surgical procedures carried out in ENT surgery. The primary indication is to correct septal deviation which is resulting in significant nasal airway obstruction. However it is often carried out to improve surgical access as part of ESS [9, 11].

Although a low risk procedure, complications may include mild post-operative bleeding to more significant septal perforation leading to whistling and saddle nose deformity, septal abscess, and numbness or sensitivity of the upper teeth or lip [9, 12, 13].

In Ireland in 2017, there were 687 septoplasties and 487 ESS performed [14]. There are seemingly no rates published detailing the rates of concurrent septoplasty with ESS in Ireland. The rates of concurrent septoplasty with ESS vary considerable in published literatures. Statistics range from 19.5 to 27% of ESS cases including concurrent septoplasty [7, 15].

Considering the high rates of concurrent septoplasty in ESS published in the literature and the lack of published rates of concurrent septoplasty in Ireland, we wished to identify these statistics in our centre. In addition, we wished to gain an insight into the indications of ESS and for concurrent septoplasty and the method by which the requirement for septoplasty was identified.

## Methods

A retrospective cohort study was performed following review at the Research and Ethics committee. Inclusion criteria for this study considered all patients who underwent ESS procedures for all indications in The Mater Misericordiae University Hospital (MMUH) by two ENT consultants between 01/01/2022 and 31/12/2023.

These patients were subsequently analysed to identify those who underwent concurrent septoplasty using their post-operative notes. Of those whom underwent concurrent septoplasty, their charts were analysed to identify if septoplasty was identified as a likely requirement prior to surgery in the outpatient department (OPD) and subsequently consented for the procedure. Pre-operative Investigations for these patients such as CT and nasoendoscopy was also noted. Other factors such as the patients diagnosis and if patients had revision surgery was noted.

Primary endpoints included the frequency and indications of concurrent septoplasty. Secondary outcomes included the indications for ESS and to determine within the revision cohort the frequency of septoplasty performed.

Patients were excluded if they had a documented previous ESS or septoplasty in MMUH prior to the study period or documented in their post-operative notes.

Patient demographics were documented.

## Results

### Demographics

In total, 90 cases (84 patients with 6 revisions) fit the inclusion criteria. The mean (SD) age was 51.9 (17.02) years. There were 56 males and 28 females.

### Diagnosis

Indications for surgery were primarily CRS in 70 patients. Of these, 52 had nasal polyps. Eight cases involved tumour resection and, 6 involved a biopsy, 4 were mucoceles, and 2 for inverted papilloma (Table 1).

**Table 1:** Indications for ESS

Indication for ESS	Frequency
CRS with nasal polyps	52
CRS without nasal polyps	18
Tumour resection	8
Biopsy	6
Mucocele	4
Inverted papilloma	2

### Septoplasty frequency

Six patients had a concurrent septoplasty performed at the time of their ESS (Table 2). All patients whom underwent concurrent septoplasty had CRS (4 with nasal polyps and 2 without). Three had obstructive septal spurs and 3 had obstructive deviated septum as the indication for septoplasty (Table 3).

**Table 2:** Frequency of ESS with concurrent septoplasty

Surgery	Frequency
Number of patients identified	84
Total ESS procedures	90
ESS with concurrent septoplasty	6 (6/90 × 100 = 6.7%)

**Table 3:** Indications for concurrent septoplasty

Indications for concurrent septoplasty	Frequency
Obstructive septal spur	3
Obstructive septal deviation	3

### Pre-operative planning

The 6 patients that required concurrent septoplasty were all identified in the clinic. These patients were counselled of the benefits and risks of concurrent septoplasty.

### Revision surgery

Six patients had revision surgery during the time period, and five had single revisions with one patient having had two revisions. The indications for revision surgery are outlined in Table 4.

**Table 4:** Revision ESS indications

Patient	Initial ESS indication	First revision	Secondary revision
A	CRS with nasal polyps	Tumour resection	Tumour resection
B	Mucocele	Mucolcele	
C	CRS	CRS	
D	Biopsy	Mass	
E	Inverted papiloma	CRS with nasal polyps	

## Discussion

This study was performed to audit the increasingly popular surgical approach of ESS to evaluate the proportion that required concurrent septoplasty for surgical access. In our study group, we found that the rates for concurrent septoplasty (6.7%) are significantly lower that what has been previously published in international journals (19.5–27%). This is encouraging as it may reduce theatre time and complications of septoplasty.

In the centre’s OPD, all patients being considered for ESS will have had a flexible nasoendoscopy to evaluate the accessibility of the nasal cavity. Visualisation of the axilla of the middle turbinate is a useful landmark to predict the need for concurrent septoplasty. These patients will be informed and subsequently consented for concurrent septoplasty. This process has allowed us to correctly identify patients requiring concurrent septoplasty and prevents the needs for blanket consenting.

Technological advances likely contribute to the reduction of concurrent septoplasty. The advances over the last several decades have been with size reduction in endoscopes, improvement in angled instruments and microdebriders. Moreover, the reduction in size of endoscopes appears to have greater utility at identifying structures of the nasal cavity and is more comfortable for the surgeon [16, 17]. Pre-operative CT sinus is also recommended as best practice with CT-guided interactive imaging is also used in many centres. Although published 14 and 18 years ago respectively, *Govindaraj *et al*.* and *Kennedy* provides a detailed account on the technical innovations used in ESS outlining the advantages of the technical innovations which have occurred over the years [18, 19].

Surgical skill is a known contributing factor and may have contributed significantly. The two consultant surgeons whom completed the cases are both experienced ENT surgeons with special interest in rhinology.

The different indications for ESS are unlikely to be surprising to most working in rhinology. CRS with and without nasal polyposis is a common for ESS and was the most common indication for ESS in our centre. The other indications demonstrated illustrate the variety of disease that may be treated which is no longer confined to CRS.

While we interrogated the charts of those whom had concurrent septoplasty for consent, we did not evaluate the charts of those whom were identified as likely needing concurrent septoplasty and subsequently did not. This would have been interesting to further evaluate the success rate of preoperative nasoendoscopy and CT. The predictive value of these investigations for determining septoplasty may have been determined in this study.

## Limitations

This study was a single-centre retrospective study with a relatively small sample size. Moreover it is difficult to determine if this centre is representative of other national rates and so a multi-centre study would be of benefit.

## Conclusion

In our series, we have found the rate of ESS with concurrent septoplasty in our centre to be lower than what was published in international journals and may be used as a benchmark in Ireland for which no rates could be found.

We have also found that preoperative nasoendoscopy is an effective way of identifying the requirement for improved surgical access during ESS and thus those that will likely require septoplasty.

## Data Availability

Available on request.
